# Hearing preservation and facial nerve outcomes in vestibular schwannoma surgery: total removal using the retrosigmoid approach

**Published:** 2012-11

**Authors:** Masoud Shirvani, Mohammad Ali Arami

**Affiliations:** ^*a*^Department of Neurosurgery, Milad Hospital , Tehran, Iran.

## Abstract

**Background:**

The present study aims to report the results and the prognostic and technical factors for hearing preservation during removal of vestibular schwannoma by a retrosigmoid approach.

**Methods:**

This was a retrospective study conducting on the 286 vestibular showannomas removal surgeries using retrosigmoid approach during October 2007 to 2011.Preoperative and postoperative clinical, audiometric, and imaging data were collected and analyzed. The mean follow-up period was 3 years. Intraoperative neuromonitoring interventions were done for all patients and the complete excision of tumor was established by post-operative magnetic resonance imaging (MRI).

**Results:**

Functional hearing (less than 50 dB PTA and greater than50% speech discrimination) was preserved in 7 of 16 patients (43.7%) with grade II tumors, whereas only 11 of 72 patients (15.3%) with grade III tumors retained their functional hearing. Fifteen out of 16 patients (93.7%) with grade II tumors achieved a House-Brackmann (HB) grade I-III, while 63 of 72 patients (87%) with grade III tumors retained HB I-III facial function. Our result in grade IV tumors was excellence in facial nerve preservation (76%) but hearing preservation in the large tumors was only about 1%.

**Conclusions:**

Findings of the present study showed that hearing preservation depends on the tumor size and using monitoring and other modern instruments and technical skills preservation rate of it could be higher than before. Overall facial nerve preservation was excellent with greater than 90% achieving HB 1 to 3 function at final follow-up.

**Keywords:**

Hearing preservation, Facial nerve, Retrosigmoid approach


					Volume 4, Suppl. 1 Nov 2012
Publisher: Kermanshah University of Medical Sciences
URL: http://www.jivresearch.org
Copyright:
Congress of Iranian Neurosurgeons, 14-16 November 2012, Kermanshah, Iran
Paper No. 20
Hearing preservation and facial nerve outcomes in vestibular
schwannoma surgery: total removal using the retrosigmoid
approach
Masoud Shirvani a,*
, Mohammad Ali Arami a
a
Dpartment of Neurosurgery, Milad Hospital , Tehran, Iran.
Abstract:
Background: The present study aims to report the results and the prognostic and technical factors for hearing
preservation during removal of vestibular schwannoma by a retrosigmoid approach.
Methods: this was a retrospective study conducting on the 286 vestibular showannomas removal surgeries using
retrosigmoid approach during October 2007 to 2011.Preoperative and postoperative clinical, audiometric, and
imaging data were collected and analyzed. The mean follow-up period was 3 years. Intraoperative neuromonitoring
interventions were done for all patients and the complete excision of tumor was established by post-operative
magnetic resonance imaging (MRI).
Results: Functional hearing (less than 50 dB PTA and greater than50% speech discrimination) was preserved in 7 of
16 patients (43.7%) with grade II tumors, whereas only 11 of 72 patients (15.3%) with grade III tumors retained
their functional hearing. Fifteen out of 16 patients (93.7%) with grade II tumors achieved a House-Brackmann (HB)
grade I-III, while 63 of 72 patients (87%) with grade III tumors retained HB I-III facial function. Our result in grade IV
tumors was excellence in facial nerve preservation (76%) but hearing preservation in the large tumors was only
about 1%.
Conclusions: Findings of the present study showed that hearing preservation depends on the tumor size and using
monitoring and other modern instruments and technical skills preservation rate of it could be higher than before.
Overall facial nerve preservation was excellent with greater than 90% achieving HB 1 to 3 function at final follow-
up.
Keywords:
Hearing preservation, Facial nerve, Retrosigmoid approach
* Corresponding Author at:
Masoud Shirvani: PhD of Neurosurgery, Milad Hospital,Shahrake Gharb, Saif Street,Val 15, N928, Building 4, Tehran, Iran, Tel: 09121398002,
Email: m_shirvani@yahoo.com, (Shirvani M.).

				

